# Genetic characteristics and virulence of *Listeria monocytogenes* isolated from fresh vegetables in China

**DOI:** 10.1186/s12866-019-1488-5

**Published:** 2019-06-03

**Authors:** Moutong Chen, Yuetao Chen, Qingping Wu, Jumei Zhang, Jianheng Cheng, Fan Li, Haiyan Zeng, Tao Lei, Rui Pang, Qinghua Ye, Jianling Bai, Juan Wang, Xianhu Wei, Youxiong Zhang, Yu Ding

**Affiliations:** 1 Guangdong Institute of Microbiology, State Key Laboratory of Applied Microbiology Southern China, Guangdong Provincial Key Laboratory of Microbial Culture Collection and Application, Guangdong Open Laboratory of Applied Microbiology, 58# Building, 100# Yard, XianlieZhong Road, Yuexiu District, Guangzhou, 510070 Guangdong Province People’s Republic of China; 2 College of Food Science, South China Agricultural University, Guangzhou, China; 3 Department of Food Science and Technology, Jinan University, Guangzhou, China

**Keywords:** *Listeria monocytogenes*, Premature stop codons, Multilocus sequence typing, Antimicrobial resistance, Vegetable

## Abstract

**Background:**

Ready-to-eat (RTE) vegetables have become increasingly popular along with the trend of moving towards a healthy lifestyle. However, RTE vegetables are at a higher risk of containing pathogens, maybe owing to lack of rigorous sanitization procedures. To understand the prevalence and potential risk of *Listeria monocytogenes* in RTE vegetables, we investigated the contamination level and characteristics of *L. monocytogenes* isolated from fresh vegetables.

**Results:**

Twenty-three (5.49%) of the 419 vegetables samples were positive for *L. monocytogenes*. Phylogenetic group I.1 (1/2a-3a) and II.2 (1/2b-3b-7) strains were predominant in 30 isolates, which accounted for 33.3 and 50.0%, respectively. Multilocus sequence typing of the 30 isolates grouped them into nine sequence types (STs). The most common STs were ST87 (36.7%) and ST8 (26.7%). Virulence analysis showed that all 30 isolates harbored eight classical virulence genes, 10.0% isolates harbored the *llsX* gene (ST3 and ST1 strains), and 36.7% carried the *ptsA* gene and belonged to ST87. Approximately 83.3% isolates carried full-length *inlA*, whereas five isolates had premature stop codons in *inlA*, three of which belonged to ST9 and two to ST8. Antibiotic susceptibility showed the isolates were varyingly resistant to 13 antibiotics, 26.7% of the isolates were multi-drug resistant.

**Conclusions:**

The fresh vegetables contain some potential hypervirulent *L. monocytogenes* (ST1 and ST87) in the Chinese markets. In addition, the high rate of *L. monocytogenes* isolates was multi-drug resistant. Fresh raw vegetables may be a possible transmission route for *L. monocytogenes* infection in consumers. Therefore, sanitization of raw fresh vegetables should be strengthened to ensure their microbiological safety when used as RTE vegetables.

**Electronic supplementary material:**

The online version of this article (10.1186/s12866-019-1488-5) contains supplementary material, which is available to authorized users.

## Background

In recent years, there is an increasing demand for ready-to-eat (RTE) vegetables because people have realized the importance of a healthy diet and healthy lifestyle. However, the increasing consumption of fresh produce, including fruits and vegetables was recognized as a source of microbiological foodborne outbreaks in many parts of the world [1, 2].

*Listeria monocytogenes* is a common Gram-positive facultative anaerobic bacillus that is recognized as one of four foodborne pathogens by the World Health Organization. It can invasively infect humans and animals, causing severe listeriosis leading to meningoencephalitis, sepsis and fetal infection or miscarriage in pregnant women, with a mortality rate of 20–30% [3]. High-risk groups include pregnant women, infants, immunodeficient patients and the elderly [3]. Fresh vegetables and herbs, such as coriander, lettuce, tomato, cucumber, cabbage, carrot, and shallot, are generally used as raw material in RTE vegetables after minimal processing to preserve nutrients, which is an important source of RTE vegetable pollution. In recent years, reports show an increase in the prevalence of *L. monocytogenes* in fresh and RTE vegetables. A study revealed that *L. monocytogenes* was detected in 6.67% fruit and vegetable products from 2011 to 2015 in Jilin Province, China (Yang et al., 2017). Both in Europe and USA, a link between foodborne disease outbreaks and pathogen (such as *L. monocytogenes*) contaminated green leafy vegetables, such as lettuce and spinach, and their RTE salads has been reported [4]. In recent years, the occurrence of *L. monocytogenes* in vegetables has been reported in several countries, with the detection rate ranging from 0.6 to 9.1% [5–8]. China is the largest vegetable producer and consumer in the world, with an output volume of vegetables and processed products of 10.1 million tons in 2015 [9], However, there is no uniform method for risk assessment and management of *L. monocytogenes* in RTE vegetables [10],which are mostly consumed raw or after minimal processing and pathogen contamination poses a health risk.

It is critical to evaluate the virulence of *L. monocytogenes* in fresh vegetables. This study aimed to evaluate the qualitative and quantitative contamination level by *L. monocytogenes* in fresh vegetables from Chinese markets. We determined the phylogenetic group, virulence profiles, and antimicrobial resistance and performed multilocus sequence typing (MLST) to comprehensively study the virulence of *L. monocytogenes* isolated from fresh vegetables across China.

## Results

### Qualitative and quantitative analysis of *L. monocytogenes* in fresh vegetable samples

A total of 23 (5.49%) positive samples were detected by quantitative and qualitative methods in the 419 collected vegetable samples. According to the different types of vegetables collected, the positive rate of lettuce was highest (7.78%), followed by coriander (4.49%), tomato (4.90%) and cucumber (4.88%) (Table 1). Among the 23 positive samples, quantitative detection by MPN method showed that the contamination level of two samples corresponding to lettuce and tomato were up to 110 MPN/g, one tomato sample was 24 MPN/g, and the remaining 20 samples were between 0.3–10 MPN/g. We also isolated 30 strains of *L. monocytogenes* from the 23 positive samples (Additional file 1: Table S2).

**Table 1:** *Listeria monocytogenes* isolates from different vegetable samples

Types of samples	Number of samples	No. (%) of positive samples	Isolated strains number
Coriander	89	4 (4.49)	5
Lettuce	90	7 (7.78)	10
Tomato	102	5 (4.90)	7
Cucumber	123	6 (4.88)	7
Others	15	1 (6.67)	1
Total	419	23 (5.49)	30

### Phylogenetic group analysis

**Fig. 1:**
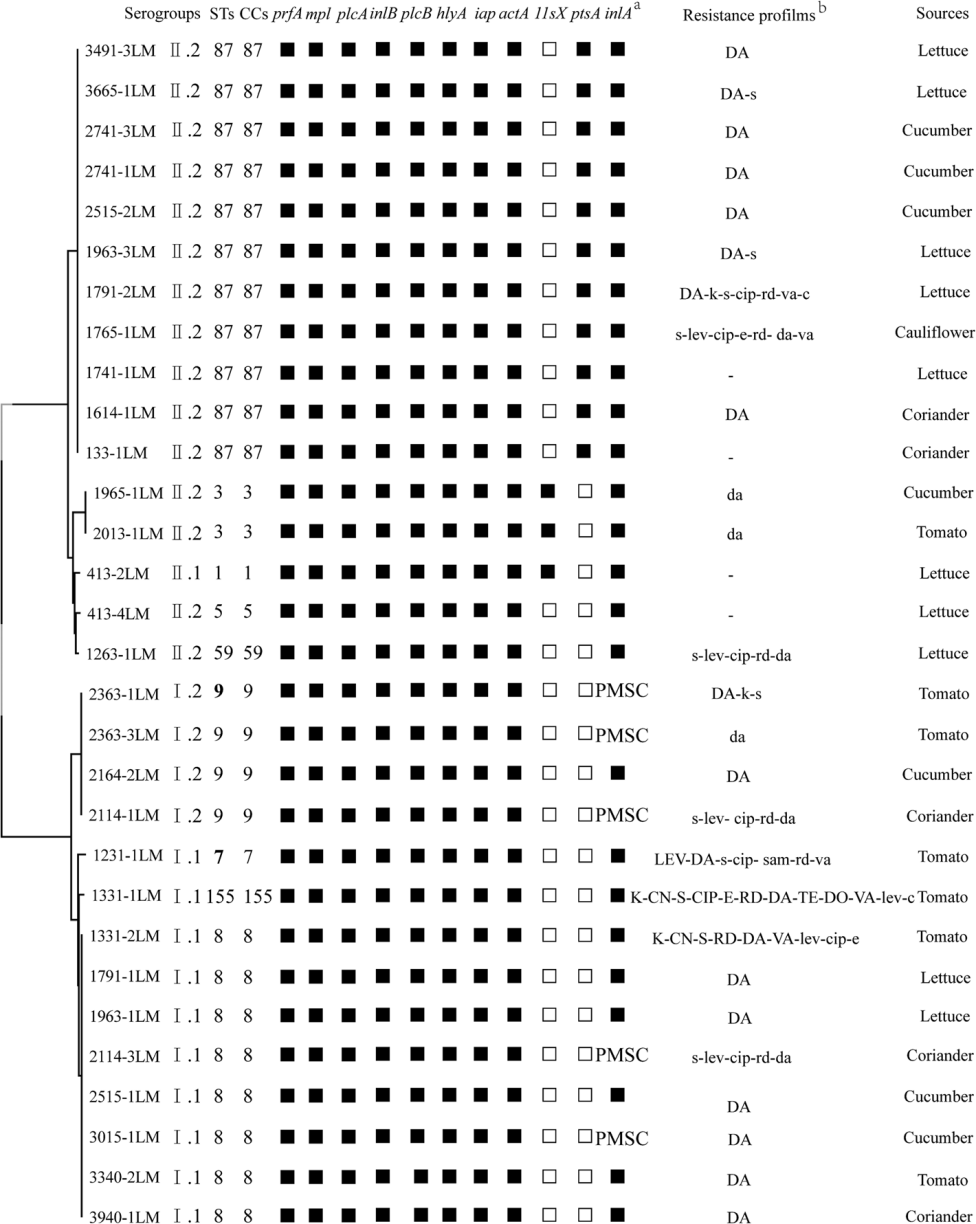
Characteristics of 30 *L. monocytogenes* isolated from fresh vegetables. Of the 10 virulence genes (*prfA*, *mpl*, *plcA*, *inlB*, *plcA*, *hly*, *iap*, *actA*, *llsX* and *ptsA*), black squares indicate the presence of the corresponding gene; white squares represent lack of corresponding gene. (a) PMSC, premature stop codons in *inlA*; black squares indicate the presence of full-length *inlA*. (b) K (k), kanamycin; CN (cn), gentamycin; S (s), streptomycin; LEV (lev), levofloxacin; CIP (cip), ciprofloxacin; SAM (sam), sulbactam/ampicillin; E (e), erythromycin; RD (rd), rifampin; DA (da), clindamycin; TE (te), tetracycline; DO (do), doxycycline; VA (va), vancomycin; C (c), chloramphenicol; − indicates no resistance. Antibiotic abbreviations in uppercase indicateresistance, while those in lowercase indicate intermediate resistance

Multiple PCR was used to analyze the phylogenetic group of 30 isolates obtained from 23 *L. monocytogenes-*positive samples (Additional file 1: Figure S1). Isolates belonging to different phylogenetic group from the same sample were included in this study. Isolates were divided into four phylogenetic groups, among which phylogenetic group I.1 (1/2a-3a) accounted for 33.3% (10/30), phylogenetic group I.2 (1/2c-3c) accounted for 13.3% (4/30), phylogenetic group II.1 (4b-4d-4e) accounted for 3.3% (1/30), and phylogenetic group II.2 (1/2b-3b-7) was predominant at 50.0% (15/30) (Fig. 1). *L. monocytogenes* of two different phylogenetic groups were isolated from three lettuce samples, one cucumber sample and one coriander sample, suggesting that there were different serotypes of *L. monocytogenes* contaminating the same vegetable sample.

### Multilocus sequence typing analysis

According to the sequencing results of the seven housekeeping genes using MLST, the sequence types (STs) and clonal complexes (CCs) of the isolates were obtained. The 30 isolates were classified into nine STs belonging to nine CCs (Fig. 1). ST87/CC87 and ST8/CC8 were predominant and accounted for 36.7% (11/30) and 26.7% (8/30), respectively. CC9 accounted for 13.3% (4/30), CC3 for 6.7% (2/30), and CC1, CC5, CC7, CC59 and CC155 were 3.3% (1/30) each, respectively.

### Virulence profiles analysis

In this study, a total of 10 virulence genes (*prfA*, *mpl*, *plcA*, *inlB*, *plcA*, *hly*, *iap*, *actA*, *llsX* and *ptsA*) of 30 isolates were detected by PCR. The results showed that except for the *llsX* and *ptsA*, all isolates carried the other eight virulence genes. Only three isolates (413-2LM, 1965-1LM and 2013-1LM) carried the *llsX* gene, which belonged to ST1 and ST3. A total of 11 (36.7%) isolates harbored the *ptsA* gene and all belonged to ST87 (Additional file 1: Figure S2). Analysis of *inlA* DNA sequencing showed that five isolates harbored PMSCs in the *inlA* gene. Four isolates (2114-1LM, 2114-3LM, 2363-1LM and 2363-3LM) had a deletion of an adenine at position 1637 of *inlA* [11], and one isolate (3015-1LM) had a deletion of an adenine at position 12 [12], These frame shift mutations lead to the creation of a nonsense codon, TAA, at position 1729 and 25, respectively. Moreover, three isolates (2114-1LM, 2363-1LM, and 2363-3LM) belonged to ST9, and the other two (3015-1LM and 2114-3LM) belonged to ST8.

### Antimicrobial resistance analysis

According to CLSI guidelines and MIC method, the antimicrobial resistance analysis of 16 antibiotic agents against the *L. monocytogenes* isolates is shown in Table 2 and Fig. 1. Among the 30 *L. monocytogenes* isolates, all strains were susceptible to three antibiotics, penicillin, ampicillin, and sulfamethoxazole/trimethoprim. In contrast, resistance to clindamycin was the most common resistance and was identified in 63.3% isolates. On the other hand, only four (13.3%) isolates (133-1LM, 413-2LM, 413-4LM, and 1741-1LM) were susceptible to all the 16 antibiotics. Other isolates showed varying degrees of resistance and/or intermediate resistance to individual antibiotics. It is worth mentioning that 26.7% isolates were defined as multi-drug resistant, of which 1331-1LM and 1331-2LM were isolated from the same tomato sample and showed resistant/intermediate resistant to 12 and 9 antibiotics, respectively.

**Table 2:** Antibiotic resistances of *Listeria monocytogenes* isolates from raw fresh vegetable samples

Antimicrobial class	Antimicrobial agent (disk content, μg or U)	Critical value (mm)			Sample number(%)		
		S	I	R	S	I	R
Aminoglycosides	Kanamycin(30)	≥18	14–17	≤13	26 (86.7)	2 (6.7)	2 (6.7)
	Gentamicin(10)	≥15	13–14	≤12	28 (93.3)	0	2 (6.7)
	Streptomycin(10)	≥15	12–14	≤11	19 (63.3)	9 (30)	2 (6.7)
Fluoroquinolones	Levofloxacin(5)	≥19	16–18	≤15	28 (93.3)	2 (6.7)	0
	Ciprofloxacin(5)	≥21	16–20	≤15	22 (73.3)	7 (23.3)	1 (3.3)
Folate pathway inhibitors	Sulfamethoxazole /trimethoprim (23.75/1.25)	≥16	11–15	≤10	30 (100)	0	0
β-lactam group	Sulbactam/ampicilin(10/10)	≥15	12–14	≤11	29 (96.7)	1 (3.3)	0
Macrolides	Erythromycin(15)	≥23	14–22	≤13	27 (90)	2 (6.7)	1 (3.3)
Ansamycins	Rifampin(5)	≥20	17–19	≤16	22 (73.3)	6 (20)	2 (6.7)
Lincosamides	Clindamycin(2)	≥21	15–20	≤14	4 (13.3)	7 (23.3)	19 (63.3)
Tetracyclines	Tetracycline(30)	≥19	15–18	≤14	29 (96.7)	0	1 (3.3)
	Doxycycline(30)	≥16	13–15	≤12	29 (96.7)	0	1 (3.3)
Glycopeptides	Vancomycin^a^(30)	≥17	15–16	≤14	25 (83.3)	3 (10)	2 (6.7)
Phenicols	Chloramphenicol (30)	≥18	13–17	≤12	28 (93.3)	2 (6.7)	0
β-Lactam	Ampicillin	<2 μg/mL	–	≥2 μg/mL	30 (100.0)	0 (0)	0 (0)
β-Lactam	Penicillin	<2 μg/mL	–	≥2 μg/mL	30 (100.0)	0 (0)	0 (0)

^a^: The critical values of *Enterococcus*; the critical values of *Staphylococcus*; *S* susceptible, *I* intermediate, *R* resistant

## Discussion

There is no doubt that *L. monocytogenes* is a critical foodborne pathogen worldwide. At present, there are very little data on the identification of *L. monocytogenes* contamination in fresh vegetables in China, especially for quantitative analysis, which limits risk assessment and the development of relevant safety standards for fresh vegetables used in RTE vegetables. In this study, 419 commercially available fresh vegetables in 43 representative cities/regions in China were investigated, of which 23 (5.49%) were positive for *L. monocytogenes* (Table 1). The highest contamination rate (7.78%) was found in lettuce sample and the MPN value of one positive sample exceeded 100 MPN/g. This could be due to the relatively large leaf surface of lettuce that is close to the soil surface and therefore easily contaminated by soil and environmental water. Wang et al. analyzed 153 RTE vegetable samples from six districts in Zigong City, Sichuan Province of China, and found a *L. monocytogenes* positive rate of 6.5% [13], which was consistent with our results. Kuanet al. reported that *L. monocytogenes* was more frequently observed in organic (9.1%) than in conventional (2.7%) vegetables in Malaysia [6]. These studies have reported different degrees of *L. monocytogenes* contamination in RTE vegetables. To date, National Health Commission of P. R. China has formulated a zero-tolerance policy for *L. monocytogenes* in cooked meat and ready-to-eat fresh meat. European Union has formulated a < 100 CFU/g policy for ready-to-eat foods during their shelf-life. However, there is no specific quantitative rule for the presence of *L. monocytogenes* in ready-to-eat vegetables. Although the prevalence of *L. monocytogenes* on fresh vegetables is low, RTE vegetables are generally not treated at high temperature, and if the raw material is contaminated, it poses a major potential transmission route of *L. monocytogenes* infection. It is necessary to perform the risk assessment of *L. monocytogenes* in vegetables for providing basic data to formulate the quantitative rule.

*L. monocytogenes* was divided into 13 serotypes, which were further divided into five phylogenetic groups by multiplex PCR [14]. In this study, *L. monocytogenes* phylogenetic group I.1 (33.3%) and II.2 (50.0%) were predominant. Previous studies have shown that phylogenetic group I.1 (1/2a-3a) of the food isolate was mainly serotype 1/2a, and phylogenetic group II.2 (1/2b-3b-7) was primarily serotype 1/2b [14]. This indicated that phylogenetic groups I.1 and II.2 of the isolates predominate in fresh vegetables, which is consistent with previous studies [15]. Of the 13 serotypes, more than 95% of disease and food contamination were associated with 4 serotypes (1/2a, 1/2b, 1/2c, and 4b), and approximately 50% of clinical cases were of serotype 4b, followed by 1/2a (27%). The vast majority of *L. monocytogenes* serotypes isolated from fresh vegetables were consistent with the serotypes of clinical pathogenic and food contaminating strains, suggesting that contamination of fresh vegetables used to make RTE vegetables may be a transmission route for *L. monocytogenes* infection.

Previous studies have shown that *L. monocytogenes* was a highly heterogeneous species with regards to pathogenicity, which consisted of different virulence clones, and clones CC1, CC2, CC4 and CC6 were strongly associated with a clinical origin. Especially, CC4 strain carried LIPI-4, a locus involves in neural and placental infection, is considered as high pathogenicity [16]. We analyzed the MLST data of 30 strains that were collected from fresh vegetables in China and observed that the frequency distribution of these clones was highly uneven (Fig. 1). The most prevalent clones were CC87 (36.7%), CC8 (26.7%), CC9, (13.3%), CC3 (6.7%), and CC1, CC5, CC7, CC59, and CC155 at 3.3%. In western countries, ST121, ST9, and ST8 are predominant in food items and food associated environments [16]. Unlike western countries, CC87 is predominant in vegetable samples in this study, which is also persistent in pre-packed smoked salmon in Singapore, Asia [17]. The PMSCs in *inlA* are mainly found in ST9 in this study, which was consistent with the results of ST121, indicating that the attenuated virulence of predominant *L. monocytogenes* STs may occur in food items and food associated environments. In addition, Wang et al. analyzed the CCs of 33 *L. monocytogenes* clinical isolates from different regions of China in the past decade, and showed that CC87 (24.2%) and CC8 (9.09%) strains were predominant [13]. These results demonstrate that *L. monocytogenes* isolated from fresh vegetables might have potentially hypervirulent. To elucidate the status of virulence genes in the isolated strains, we performed full-length sequencing of *inlA* and PCR amplification of ten other virulence genes. The results suggest that only five isolates harbored PMSCs in the *inlA* gene and all isolates harbored eight virulence genes (*prfA*, *mpl*, *plcA*, *inlB*, *plcA*, *hlyA*, *iap* and *actA*). Particularly, the *llsX* gene (encoding Listeriolysin, LLS, a hemolytic and cytotoxic factor) belonging to LIPI-3, has been greatly associated with a subset of lineage I in human listeriosis [18]. LLS acts to target the host gut microbiota, responsible for the majority of listeriosis outbreaks [19]. Four isolates (413-2LM, 1965-1LM and 2013-1LM) carried the *llsX* gene, and belonged to CC1 and CC3, indicating the presence of *Listeria* pathogenicity islands 3 (LIPI-3) in the genome, which enhances hemolytic and cytotoxic activity of *L. monocytogenes* [18]. Recent study reported that phosphotransferase system (PTS) of the cellobiose family mediates *L. monocytogenes* neural and placental tropisms, which consists of six genes (including the *ptsA* gene) [16]. A total of 11 isolates analyzed in this study carried LIPI-4 and belonged to ST87. Wang et al. reported that ST87 is the predominant clinical isolate of listeriosis in China, which is different from human infection cases in the United States and Europe [20]. In conclusion, although the *L. monocytogenes* contamination rate in fresh vegetables was low, their isolates contained phenotypes, which frequently results in listeriosis for people, including ST87, ST8 and potential high-virulence strains carrying LIPI-3. Furthermore, 83.3% isolates carried the full-length *inlA*, suggesting that fresh vegetables could become the transmission route of *L. monocytogenes* infection.

At present, the drug resistance of food-borne pathogens is a growing concern that threatens public health. Antibiotics commonly administered to treat listeriosis include penicillin, amoxicillin, ampicillin, meropenem, vancomycin, gentamicin, rifampin, cotrimoxazole, levofloxacin, and linezolid, with the first three used with the highest frequency [21]. However, *L. monocytogenes* isolates with varying levels of resistance to antibiotics has been reported. Escolar et al. found *Listeria spp.* isolated from RTE products of animal origin to have clindamycin (100%), ciprofloxacin (52%), penicillin (32%) and ampicillin (20%) resistance in Spain [22]. None of isolates was found to be resistant to ampicillin and penicillin in this study, suggesting that the first-choice drugs are still effective for listeriosis treatment. The results of this study suggest the presence of a high level of resistance to clindamycin (63.3%, see Table 2), which is used in hospital treatments [23]. On the other hand, 26.7% isolates were found to be multi-drug resistant in our study, with two strains (1331-1LM and 1331-2LM) isolated from the same tomato sample showing 110 MPN/g and being tolerant to 12 and 9 antibiotics, respectively. These results prompt the necessity of novel alternatives for antimicrobial to disrupt the emerging multidrug resistance of *L. monocytogenes*. The acquisition of multi-drug resistance is possibly related to the widespread use of antibiotics. At the same time, the bacteria can develop resistance mechanisms or acquire resistance by transmission of genetic material from other bacterial species [22]. Therefore, it is necessary and significant to further study the possible mechanism of resistance, especially the antimicrobial resistance mechanisms of *L. monocytogenes* in fresh vegetables.

## Conclusion

In conclusion, 5.49% of fresh vegetables collected from the 43 representative cities/regions markets in China were positive for *L. monocytogenes*. Phylogenetic group I.1 (33.3%) and II.2 (50.0%) of *L. monocytogenes* were dominant in fresh vegetable sources. The strains of ST87 (36.7%) and ST8 (26.7%) were predominantly identified by MLST analysis. The virulence genes *prfA*, *mpl*, *plcA*, *inlB*, *plcA*, *hlyA*, *iap* and *actA* were carried by all strains, in contrast 10.0% carried the *llsX* gene and 36.7% carried the *ptsA* gene. Moreover, 83.3% of the isolates were full-length for *inlA*, indicating that most isolates were capable of invading the host cells. Except for sulfamethoxazole, ampicillin, and penicillin, different degrees of resistance to the other 13 antibiotics was observed, and multi-drug resistant strains accounted for 26.7%. The results suggest that fresh raw vegetables may be a possible transmission vehicle for *L. monocytogenes* infection in consumers, and should be treated with rigorous sanitization treatments when used for RTE vegetable to ensure microbiological safety.

## Methods

### Sample collection

A total of 419 fresh vegetables, which form the main raw ingredients in RTE vegetables, were randomly purchased from 43 representative cities or regions of China (Additional file 1: Table S1). The collected samples included coriander (*n* = 89), lettuce (*n* = 90), tomato (*n* = 102), cucumber (*n* = 123), and other vegetables (*n* = 15). Sampling was carried out over a 5-year period (July 2011 to July 2016). After collection, all samples were immediately placed in sterile bags, kept in an insulated box with ice packs, and transported to the laboratory for analysis within 2 h after reaching the laboratory.

### Qualitative and quantitative analysis

Qualitative analysis was carried out according to the food microbiological test of *L. monocytogenes* in GB4789.40–2010 (National Food Safety Standards of China) with slight modification. In brief, samples were cut and mixed on a sterile workbench, and 25 g of each sample was homogenized in 225 mL *Listeria* enrichment broth 1 (LB1; Guangdong Huankai Co. Ltd., Guangzhou, China). Homogenates were cultured at 30 °C for 24 h, after which 0.1 mL LB1 enriched cultures were transferred to 10 mL of LB2 and incubated at 30 °C for 24 h. After incubation, the collected samples were streaked on Chromagar *Listeria* plates (Guangdong Huankai Co. Ltd., China) with an inoculation loop and incubated at 37 °C for 48 h. Three to five colonies typically blue in color with a white halo were selected for identification of *L. monocytogenes* using the Microgen ID Listeria identification system (Microgen, Camberley, United Kingdom) according to manufacturer’s instructions. The *L. monocytogenes* isolates were stored with 20% glycerol at − 40 °C for further use.

Quantitative detection of *L. monocytogenes* was performed using the operational flow of the most probable number (MPN) method [24]. 25 g of each sample was added to 225 mL of Frasher enrichment broth (Guangdong Huankai Co. Ltd., China) in a homogenization bag. The sample was shaken evenly, before transferring 10 mL (1 g), 1 mL (0.1 g), or 0.1 mL (0.01 g) of the homogenates to sterile tubes. To the latter two tubes, 10 mL Fraser broth was also added, and each dilution was prepared in triplicate [25]. After incubating at 30 °C for 48 h, the darkened Fraser tubes were selected and streaked onto *Listeria* chromogenic agar plates. After further purification, the isolates were identified using the Microgen ID Listeria identification system and multiplex PCR, and the MPN was calculated by referring to the MPN table.

### Phylogenetic group analysis

The *L. monocytogenes* isolates of fresh vegetables were identified for phylogenetic groups by multiplex PCR according to the method of Doumith et al. The multiplex PCR method can classify 13 serotypes of *L. monocytogenes* into five phylogenetic groups, which are designated as I.1 (1/2a-3a), I.2 (1/2c-3c), II.1 (4b-4d-4e), II.2 (1/2b-3b-7) and III (4a-4c). The PCR products were separated by electrophoresis on a 1.5% agarose gel (containing 0.005% Goldview stain) at 100 V for 40 min, photographed using Tanon 2500 UV camera system. Images were saved in TIFF format for further analysis.

### Multilocus sequence typing (MLST) analysis

According to the analytical method established by Ragon et al. [26], the specific primers of seven housekeeping genes (*abcZ*, *bglA*, *cat*, *dapE*, *dat*, *ldh* and *lhkA*) were used for MLST using PCR amplification on isolated strains. The *ldh* primers were *ldh*F: 5′–GACAGAACAATTGGGGATGCAATG–3′ and *ldh*R: 5′–AACGCCGTAGAATGTAGCGCCT–3′ [27]. The all annealing temperatures are 52 °C (45 °C for *bglA* and 58 °C for *ldh*). The amplified products were subjected to bidirectional sequencing, and MLST analysis was performed using the *Listeria* MLST database (http://bigsdb.pasteur.fr/listeria/listeria.html) curated by the Pasteur Institute of France. Based on MLST’s seven housekeeping genes (*abcZ*, *bglA*, *cat*, *dapE*, *dat*, *ldh* and *lhkA*) linkage sequences, the Neighbor-Joining phylogenetic tree was constructed using MEGA 7.0.

### Virulence profiles analysis

PCR amplification was performed to detect potential virulence-related genes of 30 *L. monocytogenes* isolates from fresh vegetable sources. The 10 pairs of primers and reaction conditions are shown respectively in Table 3. Multiple naturally occurring mutations leading to a premature stop codon (PMSC) in *inlA* have been reported worldwide, and these mutations were causally associated with attenuated virulence [28, 29]. Therefore, the *inlA* gene was investigated by amplicon sequencing to determine the presence of PMSCs. The full-length *inlA* gene (2403 bp) was amplified from 30 isolates using external primers, and internal primers were used for sequencing [30]. The *inlA* sequences were assembled with MEGA software (version7.0.26). By comparing to the complete *inlA* sequence of the *L. monocytogenes* EGDe reference strain, sites of PMSC mutations in *inlA* were determined [31].

**Table 3:** Primers used for virulence genes detection of *Listeria monocytogenes* strains

Genes	Sequences (5′-3′)	Tm (°C)	Length (bp)	Reference
*prfA*	CTGTTGGAGCTCTTCTTGGTGAAGCAATCG	60	1060	[35]
	AGCAACCTCGGTACCATATACTAACTC			
*mpl*	ATAGCTTTTCAGGCTCATTTCA	60	1184	this study
	ATAGCTTTTCAGGCTCATTTCA			
*plcA*	CTGCTTGAGCGTTCATGTCTCATCCCCC	60	1484	[35]
	CATGGGTTTCACTCTCCTTCTAC			
*inlB*	GATATTGTGCCACTTTCAGGT	60	367	[36]
	CCTCTTTCAGTGGTTGGGT			
*plcA*	CTGCTTGAGCGTTCATGTCTCATCCCCC	60	436	[37]
	CATGGGTTTCACTCTCCTTCTAC			
*hly*	CTTGCAACTGCTCTTTAGTAACAGC	60	706	[38]
	ACAAGCTGCACCTGTTGCAG			
*iap*	ACAAGCTGCACCTGTTGCAG	60	131	[39]
	TGACAGCGTGTGTAGTAGCA			
*actA*	CGCCGCGGAAATTAAAAAAAGA	60	839	[40]
	ACGAAGGAACCGGGCTGCTAG			
*llxS*	TTATTGCATCAATTGTTCTAGGG	52	200	[41]
	CCCCTATAAACATCATGCTAGTG			
*ptsA* ^*a*^	TCCTTTTTCTTTGTTGCGGA	52	450	[13]
	TCTGAAGCTGTACGAAGACA			

^a^ Tm (°C) with slight modification

### Antimicrobial resistance analysis

According to the criteria of *Staphylococcus* or *Enterococcus* susceptibility test of the Clinical and Laboratory Standards Institute (CLSI) guidelines (M100-27th ed.) [32], all strains were analyzed by the disk diffusion method. A total of 14 antibiotic agents (classified in 10 categories) were tested at specific concentrations per disk. Details are provided in Table 2. *Staphylococcus aureus* ATCC 25923 and *Escherichia coli* ATCC 25922 were used as quality control strains. Particularly, ampicillin and penicillin were tested by minimal inhibitory concentration (MIC) method. Isolates showing growth in wells with a concentration more than or equal to 2 μg/mL were considered resistant strains according to Standards Institute guidelines [33]. Isolates which resistant to three or more types of antibiotic resistance were defined as multidrug-resistant strains [34].

## Additional file

Additional file 1: **Table S1.** The detail detection results of *Listeria monocytogenes* in 419 vegetable samples. **Table S2.** *Listeria monocytogenes* strains isolated from fresh vegetables **Figure S1.** Serogroup analysis of *Listeria monocytogenes* strains isolated from fresh vegetable samples by multiplex PCR. The strain no. 1–30 correspond to Table S2. **Figure S2.** The presence of virulence-related genes in *Listeria monocytogenes* isolated from fresh vegetable samples. A, *prfA*; B, *mpl*; C, *plcA*; D, *inlB*; E, *plcA*; F, *hly*; G, *iap*; H, *actA*; I, *llsX*; J, *ptsA*.*:The strain no. 1–30 correspond to Additional file 1: Table S2. (ZIP 8979 kb)

## Abbreviations

CCs: Clonal complexes
CLSI: Clinical and Laboratory Standards Institute
*L. monocytogenes*: *Listeria monocytogenes*
LB1: *Listeria* enrichment broth 1
LB2: *Listeria* enrichment broth 2
MIC: Minimal inhibitory concentration
MLST: Multilocus sequence typing
MPN: Most probable number
PMSC: Premature stop codon
RTE: Ready-to-eat
STs: Sequence types
