# Supplementary material for: Genetic characteristics and virulence of Listeria monocytogenes isolated from fresh vegetables in China
Source: BMC Microbiol. 2019 Jun 3;19:119. doi: 10.1186/s12866-019-1488-5 (PMC6547522; doi:10.1186/s12866-019-1488-5)
Supplement: Supplementary file 1 — Table S1. The detail detection results of Listeria monocytogenes in 419 vegetable samples. Table S2. Listeria monocytogenes strains isolated from fresh vegetables Figure S1. Serogroup analysis of Listeria monocytogenes strains isolated from fresh vegetable samples by multiplex PCR. The strain no. 1–30 correspond to Table S2. Figure S2. The presence of virulence-related genes in Listeria monocytogenes isolated from fresh vegetable samples. A, prfA; B, mpl; C, plcA; D, inlB; E, plcA; F, hly; G, iap; H, actA; I, llsX; J, ptsA.*:The strain no. 1–30 correspond to Additional file 1: Table S2. (ZIP 8979 kb) [file 12866_2019_1488_MOESM1_ESM.zip › Additional files.doc]

**Additional file 1**

**Table S1 The detail detection results of *Listeria monocytogenes* in 419 vegetable samples**

| Number | Name of Sample | Samples | Collect city | Date | Detection results | | |
| --- | --- | --- | --- | --- | --- | --- | --- |
|  |  |  |  |  | Qualitative results | Quantitative results | Isolates |
|  |  |  |  |  |  |  |  |
| 1 | YXC007 | Lettuce | Yuexiu | Jul.27.2011 | ﹣ | <0.3 |  |
| 2 | YXC014 | Water spinach | Yuexiu | Jul.27.2012 | ﹣ | <0.3 |  |
| 3 | YXC016 | Tomato | Yuexiu | Jul.27.2013 | ﹣ | <0.3 |  |
| 4 | YXJ031 | Cucumber | Yuexiu | Jul.27.2014 | ﹣ | <0.3 |  |
| 5 | YXJ032 | Coriander | Yuexiu | Jul.27.2015 | ﹣ | <0.3 |  |
| 6 | FCC050 | Cucumber | Liwan | Aug.8.2011 | ﹣ | <0.3 |  |
| 7 | FCC059 | Lettuce | Liwan | Aug.8.2011 | ﹣ | <0.3 |  |
| 8 | FCJ089 | Water spinach | Liwan | Aug.8.2011 | ﹣ | <0.3 |  |
| 9 | FCJ090 | Coriander | Liwan | Aug.8.2011 | ﹣ | <0.3 |  |
| 10 | PYC107 | Lettuce | Panyu | Aug.30.2011 | ﹣ | <0.3 |  |
| 11 | PYC108 | Tomato | Panyu | Aug.30.2011 | ﹣ | <0.3 |  |
| 12 | PYC109 | Cucumber | Panyu | Aug.30.2011 | ﹣ | <0.3 |  |
| 13 | PYJ133 | Coriander | Panyu | Aug.30.2011 | ＋ | 0.3 | 133-1LM |
| 14 | PYN138 | Water spinach | Panyu | Aug.30.2011 | ﹣ | <0.3 |  |
| 15 | CHC154 | Lettuce | Conghua | Sep.13.2011 | ﹣ | <0.3 |  |
| 16 | CHC155 | Tomato | Conghua | Sep.13.2011 | ﹣ | <0.3 |  |
| 17 | CHC165 | Cucumber | Conghua | Sep.13.2011 | ﹣ | <0.3 |  |
| 18 | CHJ181 | Coriander | Conghua | Sep.13.2011 | ﹣ | <0.3 |  |
| 19 | CHJ184 | Water spinach | Conghua | Sep.13.2011 | ﹣ | <0.3 |  |
| 20 | CHJ197 | Lettuce | Conghua | Sep.13.2011 | ﹣ | <0.3 |  |
| 21 | ZCC209 | Lettuce | Zengcheng | Sep.25.2011 | ﹣ | <0.3 |  |
| 22 | ZCC210 | Tomato | Zengcheng | Sep.25.2011 | ﹣ | <0.3 |  |
| 23 | ZCC211 | Cucumber | Zengcheng | Sep.25.2011 | ﹣ | <0.3 |  |
| 24 | ZCJ235 | Lettuce | Zengcheng | Sep.25.2011 | ﹣ | <0.3 |  |
| 25 | ZCN244 | Coriander | Zengcheng | Sep.25.2011 | ﹣ | <0.3 |  |
| 26 | ZCN249 | Water spinach | Zengcheng | Sep.25.2011 | ﹣ | <0.3 |  |
| 27 | SZC263 | Lettuce | Shenzhen | Dec.15.2011 | ﹣ | <0.3 |  |
| 28 | SZC264 | Coriander | Shenzhen | Dec.15.2011 | ﹣ | <0.3 |  |
| 29 | SZJ280 | Coriander | Shenzhen | Dec.15.2011 | ﹣ | <0.3 |  |
| 30 | SZN295 | Lettuce | Shenzhen | Dec.15.2011 | ﹣ | <0.3 |  |
| 31 | SGC313 | Lettuce | Shaoguan | Dec.22.2011 | ﹣ | <0.3 |  |
| 32 | SGC314 | Coriander | Shaoguan | Dec.22.2011 | ﹣ | <0.3 |  |
| 33 | SGJ332 | Coriander | Shaoguan | Dec.22.2011 | ﹣ | <0.3 |  |
| 34 | SGN347 | Lettuce | Shaoguan | Dec.22.2011 | ﹣ | <0.3 |  |
| 35 | ZJC363 | Lettuce | Zhanjiang | Jan.5.2012 | ﹣ | <0.3 |  |
| 36 | ZJC364 | Coriander | Zhanjiang | Jan.5.2012 | ﹣ | <0.3 |  |
| 37 | ZJJ380 | Coriander | Zhanjiang | Jan.5.2012 | ﹣ | <0.3 |  |
| 38 | ZJN395 | Lettuce | Zhanjiang | Jan.5.2012 | ﹣ | <0.3 |  |
| 39 | STC413 | Lettuce | Shantou | Feb.17.2012 | ＋ | 2.3 | 413-2LM 413-4LM |
| 40 | STC414 | Coriander | Shantou | Feb.17.2012 | ﹣ | <0.3 |  |
| 41 | STJ430 | Coriander | Shantou | Feb.17.2012 | ﹣ | <0.3 |  |
| 42 | STN445 | Lettuce | Shantou | Feb.17.2012 | ﹣ | <0.3 |  |
| 43 | HYC463 | Lettuce | Heyuan | Mar.13.2012 | ﹣ | <0.3 |  |
| 44 | HYC464 | Coriander | Heyuan | Mar.13.2012 | ﹣ | <0.3 |  |
| 45 | HYJ482 | Coriander | Heyuan | Mar.13.2012 | ﹣ | <0.3 |  |
| 46 | HYN497 | Lettuce | Heyuan | Mar.13.2012 | ﹣ | <0.3 |  |
| 47 | HKC513 | Lettuce | Haikou | May.3.2012 | ﹣ | <0.3 |  |
| 48 | HKC514 | Coriander | Haikou | May.3.2012 | ﹣ | <0.3 |  |
| 49 | HKJ530 | Coriander | Haikou | May.3.2012 | ﹣ | <0.3 |  |
| 50 | HKN545 | Lettuce | Haikou | May.3.2012 | ﹣ | <0.3 |  |
| 51 | SYC563 | Lettuce | Sanya | May.17.2012 | ﹣ | <0.3 |  |
| 52 | SYC564 | Coriander | Sanya | May.17.2012 | ﹣ | <0.3 |  |
| 53 | SYJ580 | Coriander | Sanya | May.17.2012 | ﹣ | <0.3 |  |
| 54 | SYN595 | Lettuce | Sanya | May.17.2012 | ﹣ | <0.3 |  |
| 55 | BHC613 | Lettuce | Beihai | Jun.6.2012 | ﹣ | <0.3 |  |
| 56 | BHC614 | Coriander | Beihai | Jun.6.2012 | ﹣ | <0.3 |  |
| 57 | BHJ630 | Coriander | Beihai | Jun.6.2012 | ﹣ | <0.3 |  |
| 58 | BHN645 | Lettuce | Beihai | Jun.6.2012 | ﹣ | <0.3 |  |
| 59 | NNC663 | Lettuce | Nanning | Jul.2.2012 | ﹣ | <0.3 |  |
| 60 | NNC664 | Coriander | Nanjing | Jul.2.2012 | ﹣ | <0.3 |  |
| 61 | NNJ680 | Lettuce | Nanning | Jul.2.2012 | ﹣ | <0.3 |  |
| 62 | NNN695 | Coriander | Nanjing | Jul.2.2012 | ﹣ | <0.3 |  |
| 63 | FZC713 | Lettuce | Fuzhou | Jul.18.2012 | ﹣ | <0.3 |  |
| 64 | FZC714 | Coriander | Fuzhou | Jul.18.2012 | ﹣ | <0.3 |  |
| 65 | FZJ730 | Coriander | Fuzhou | Jul.18.2012 | ﹣ | <0.3 |  |
| 66 | FZN745 | Lettuce | Fuzhou | Jul.18.2012 | ﹣ | <0.3 |  |
| 67 | XMC763 | Lettuce | Xiamen | Aug.6.2012 | ﹣ | <0.3 |  |
| 68 | XMC764 | Coriander | Xiamen | Aug.6.2012 | ﹣ | <0.3 |  |
| 69 | XMJ780 | Coriander | Xiamen | Aug.6.2012 | ﹣ | <0.3 |  |
| 70 | XMN795 | Lettuce | Xiamen | Aug.6.2012 | ﹣ | <0.3 |  |
| 71 | SHC813 | Lettuce | Shanghai | Sep.5.2012 | ﹣ | <0.3 |  |
| 72 | SHC814 | Coriander | Shanghai | Sep.5.2012 | ﹣ | <0.3 |  |
| 73 | SHC815 | vegetable salad | Shanghai | Sep.5.2012 | ﹣ | <0.3 |  |
| 74 | SHJ831 | Coriander | Shanghai | Sep.5.2012 | ﹣ | <0.3 |  |
| 75 | SHN846 | Cucumber | Shanghai | Sep.5.2012 | ﹣ | <0.3 |  |
| 76 | HFC863 | Coriander | Hefei | Sep.16.2012 | ﹣ | <0.3 |  |
| 77 | HFC864 | Coriander | Heifei | Sep.16.2012 | ﹣ | <0.3 |  |
| 78 | HFJ881 | Cucumber | Hefei | Sep.16.2012 | ﹣ | <0.3 |  |
| 79 | HFJ896 | Cucumber | Hefei | Sep.16.2012 | ﹣ | <0.3 |  |
| 80 | NCC913 | Lettuce | Nanchang | Sep.23.2012 | ﹣ | <0.3 |  |
| 81 | NCC914 | Coriander | Nanchang | Sep.23.2012 | ﹣ | <0.3 |  |
| 82 | NCC915 | Tomato | Nanchang | Sep.23.2012 | ﹣ | <0.3 |  |
| 83 | NCJ931 | Coriander | Nanchang | Sep.23.2012 | ﹣ | <0.3 |  |
| 84 | NCN946 | Cucumber | Nanchang | Sep.23.2012 | ﹣ | <0.3 |  |
| 85 | WHC963 | Lettuce | Wuhan | Oct.9.2012 | ﹣ | <0.3 |  |
| 86 | WHC964 | Coriander | Wuhan | Oct.9.2012 | ﹣ | <0.3 |  |
| 87 | WHC965 | *Crowndaisy chrysanthemum* | Wuhan | Oct.9.2012 | ﹣ | <0.3 |  |
| 88 | WHJ981 | Coriander | Wuhan | Oct.9.2012 | ﹣ | <0.3 |  |
| 89 | WHN996 | Cucumber | Wuhan | Oct.9.2012 | ﹣ | <0.3 |  |
| 90 | CDC1013 | Lettuce | Chengdu | Oct.16.2012 | ﹣ | <0.3 |  |
| 91 | CDC1014 | Coriander | Chengdu | Oct.16.2012 | ﹣ | <0.3 |  |
| 92 | CDC1015 | Tomato | Chengdu | Oct.16.2012 | ﹣ | <0.3 |  |
| 93 | CDJ1031 | Coriander | Chengdu | Oct.16.2012 | ﹣ | <0.3 |  |
| 94 | CDN1046 | Cucumber | Chengdu | Oct.16.2012 | ﹣ | <0.3 |  |
| 95 | KMC1063 | Lettuce | Kunming | Nov.4.2012 | ﹣ | <0.3 |  |
| 96 | KMC1064 | Coriander | Kunming | Nov.4.2012 | ﹣ | <0.3 |  |
| 97 | KMC1065 | Cucumber | Kunming | Nov.4.2012 | ﹣ | <0.3 |  |
| 98 | KMJ1081 | Coriander | Kunming | Nov.4.2012 | ﹣ | <0.3 |  |
| 99 | KMN1096 | White radish | Kunming | Nov.4.2012 | ﹣ | <0.3 |  |
| 100 | LZC1113 | Lettuce | Lanzhou | Nov.12.2012 | ﹣ | <0.3 |  |
| 101 | LZC1114 | Cucumber | Lanzhou | Nov.12.2012 | ﹣ | <0.3 |  |
| 102 | LZC1115 | Tomato | Lanzhou | Nov.12.2012 | ﹣ | <0.3 |  |
| 103 | LZJ1131 | Coriander | Lanzhou | Nov.12.2012 | ﹣ | <0.3 |  |
| 104 | LZN1146 | Cucumber | Lanzhou | Nov.12.2012 | ﹣ | <0.3 |  |
| 105 | HEB1163 | Lettuce | Harbin | Nov.20.2012 | ﹣ | <0.3 |  |
| 106 | HEB1164 | Cucumber | Harbin | Nov.20.2012 | ﹣ | <0.3 |  |
| 107 | HEB1165 | Coriander | Harbin | Nov.20.2012 | ﹣ | <0.3 |  |
| 108 | HEB1181 | Coriander | Harbin | Nov.20.2012 | ﹣ | <0.3 |  |
| 109 | HEB1196 | Cucumber | Harbin | Nov.20.2012 | ﹣ | <0.3 |  |
| 110 | XAC1213 | Lettuce | Xi'an | Nov.29.2012 | ﹣ | <0.3 |  |
| 111 | XAC1214 | Coriander | xi'an | Nov.29.2012 | ﹣ | <0.3 |  |
| 112 | XAC1215 | Cucumber | Xi'an | Nov.29.2012 | ﹣ | <0.3 |  |
| 113 | XAJ1231 | Tomato | Xi'an | Nov.29.2012 | ＋ | 0.3 | 1231-1LM |
| 114 | XAN1246 | Shallot | Xi'an | Nov.29.2012 | ﹣ | <0.3 |  |
| 115 | TYC1263 | Lettuce | Taiyuan | Dec.11.2012 | ＋ | 110 | 1263-1LM |
| 116 | TYC1264 | Coriander | Taiyuan | Dec.11.2012 | ﹣ | <0.3 |  |
| 117 | TYC1265 | Cucumber | Taiyuan | Dec.11.2012 | ﹣ | <0.3 |  |
| 118 | TYJ1281 | Coriander | Taiyuan | Dec.11.2012 | ﹣ | <0.3 |  |
| 119 | TYN1296 | Shallot | Taiyuan | Dec.11.2012 | ﹣ | ﹣ |  |
| 120 | BJC1313 | Lettuce | Beijing | Dec.18.2012 | ﹣ | <0.3 |  |
| 121 | BJC1314 | Coriander | Beijing | Dec.18.2012 | ﹣ | <0.3 |  |
| 122 | BJC1315 | Cucumber | Beijing | Dec.18.2012 | ﹣ | <0.3 |  |
| 123 | BJJ1331 | Tomato | Beijing | Dec.18.2012 | ＋ | 110 | 1331-1LM 1331-2LM |
| 124 | BJN1346 | Shallot | Beijing | Dec.18.2012 | ﹣ | <0.3 |  |
| 125 | JNC1363 | Lettuce | Jinan | Dec.25.2012 | ﹣ | <0.3 |  |
| 126 | JNC1364 | Coriander | jinan | Dec.25.2012 | ﹣ | <0.3 |  |
| 127 | JNC1365 | Cucumber | jinan | Dec.25.2012 | ﹣ | <0.3 |  |
| 128 | JNJ1381 | Coriander | jinan | Dec.25.2012 | ﹣ | <0.3 |  |
| 129 | JNN1396 | Lettuce | Jinan | Dec.25.2012 | ﹣ | <0.3 |  |
| 130 | YXJ1440 | Tomato | Yuexiu | Mar.4.2013 | ﹣ | <0.3 |  |
| 131 | YXC1413 | Lettuce | Yuexiu | Mar.4.2013 | ﹣ | <0.3 |  |
| 132 | YXC1414 | Coriander | Yuexiu | Mar.4.2013 | ﹣ | <0.3 |  |
| 133 | YXC1415 | Cucumber | Yuexiu | Mar.4.2013 | ﹣ | <0.3 |  |
| 134 | YXJ1441 | Cucumber | Yuexiu | Mar.4.2013 | ﹣ | <0.3 |  |
| 135 | LWC1463 | Lettuce | Liwan | Mar.12.2013 | ﹣ | <0.3 |  |
| 136 | LWC1464 | Lettuce | Liwan | Mar.12.2013 | ﹣ | <0.3 |  |
| 137 | LWC1465 | Cucumber | Liwan | Mar.12.2013 | ﹣ | <0.3 |  |
| 138 | LWN1490 | Tomato | Liwan | Mar.12.2013 | ﹣ | <0.3 |  |
| 139 | LWN1491 | Lettuce | Liwan | Mar.12.2013 | ﹣ | <0.3 |  |
| 140 | PYC1513 | Lettuce | Panyu | Mar.19.2013 | ﹣ | <0.3 |  |
| 141 | PYC1514 | Cucumber | Panyu | Mar.19.2013 | ﹣ | <0.3 |  |
| 142 | PYC1515 | Crowndaisy chrysanthemum | Panyu | Mar.19.2013 | ﹣ | <0.3 |  |
| 143 | PYN1540 | Tomato | Panyu | Mar.19.2013 | ﹣ | <0.3 |  |
| 144 | PYN1541 | Lettuce | Panyu | Mar.19.2013 | ﹣ | <0.3 |  |
| 145 | CHC1563 | Lettuce | Conghua | Mar.25.2013 | ﹣ | <0.3 |  |
| 146 | CHC1564 | Coriander | Conghua | Mar.25.2013 | ﹣ | <0.3 |  |
| 147 | CHC1565 | Cucumber | Conghua | Mar.25.2013 | ﹣ | <0.3 |  |
| 148 | CHJ1590 | Tomato | Conghua | Mar.25.2013 | ﹣ | <0.3 |  |
| 149 | CHJ1591 | Cucumber | Conghua | Mar.25.2013 | ﹣ | <0.3 |  |
| 150 | ZCC1613 | Shallot | Zengcheng | Apr.9.2013 | ﹣ | <0.3 |  |
| 151 | ZCC1614 | Coriander | Zengcheng | Apr.9.2013 | ＋ | 0.3 | 1614-1LM |
| 152 | ZCC1615 | Cucumber | Zengcheng | Apr.9.2013 | ﹣ | <0.3 |  |
| 153 | ZCJ1640 | Tomato | Zengcheng | Apr.9.2013 | ﹣ | <0.3 |  |
| 154 | ZCJ1641 | Cucumber | Zengcheng | Apr.9.2013 | ﹣ | <0.3 |  |
| 155 | SZC1663 | Tomato | Shenzhen | May.6.2013 | ﹣ | <0.3 |  |
| 156 | SZC1664 | Coriander | Shenzhen | May.6.2013 | ﹣ | <0.3 |  |
| 157 | SZC1665 | Shallot | Shenzhen | May.6.2013 | ﹣ | <0.3 |  |
| 158 | SZJ1690 | Tomato | Shenzhen | May.6.2013 | ﹣ | <0.3 |  |
| 159 | SZJ1691 | Lettuce | Shenzhen | May.6.2013 | ﹣ | <0.3 |  |
| 166 | STC1713 | Lettuce | Shantou | May.16.2013 | ﹣ | <0.3 |  |
| 167 | STC1714 | Coriander | Shantou | May.16.2013 | ﹣ | <0.3 |  |
| 168 | STC1715 | Cucumber | Shantou | May.16.2013 | ﹣ | <0.3 |  |
| 160 | STJ1740 | Tomato | Shantou | May.16.2013 | ﹣ | <0.3 |  |
| 169 | STJ1741 | Lettuce | Shantou | May.16.2013 | ＋ | 0.3 | 1741-1LM |
| 161 | ZJC1763 | Tomato | Zhanjiang | May.26.2013 | ﹣ | <0.3 |  |
| 162 | ZJC1764 | Lettuce | Zhanjiang | May.26.2013 | ﹣ | <0.3 |  |
| 163 | ZJC1765 | Shallot | Zhanjiang | May.26.2013 | ＋ | 0.3 | 1765-1LM |
| 164 | ZJJ1790 | Tomato | Zhanjiang | May.26.2013 | ﹣ | <0.3 |  |
| 165 | ZJJ1791 | Lettuce | Zhanjiang | May.26.2013 | ＋ | 3.8 | 1791-1LM 1791-2LM |
| 170 | SGC1813 | Lettuce | Shaoguan | Jun.4.2013 | ﹣ | <0.3 |  |
| 171 | SGC1814 | Coriander | Shaoguan | Jun.4.2013 | ﹣ | <0.3 |  |
| 172 | SGC1815 | Cucumber | Shaoguan | Jun.4.2013 | ﹣ | <0.3 |  |
| 173 | SGJ1840 | Tomato | Shaoguan | Jun.4.2013 | ﹣ | <0.3 |  |
| 174 | SGJ1841 | Cucumber | Shaoguan | Jun.4.2013 | ﹣ | <0.3 |  |
| 175 | HYC1863 | Lettuce | Heyuan | Jun.18.2013 | ﹣ | <0.3 |  |
| 176 | HYC1864 | Coriander | Heyuan | Jun.18.2013 | ﹣ | <0.3 |  |
| 177 | HYC1865 | Cucumber | Heyuan | Jun.18.2013 | ﹣ | <0.3 |  |
| 178 | HYJ1890 | Tomato | Heyuan | Jun.18.2013 | ﹣ | <0.3 |  |
| 179 | HYJ1891 | Cucumber | Heyuan | Jun.18.2013 | ﹣ | <0.3 |  |
| 180 | XAC1913 | Lettuce | Xi'an | Jul.11.2013 | ﹣ | <0.3 |  |
| 181 | XAC1914 | Coriander | Xi'an | Jul.11.2013 | ﹣ | <0.3 |  |
| 182 | XAC1915 | Cucumber | Xi'an | Jul.11.2013 | ﹣ | <0.3 |  |
| 183 | XAJ1940 | Tomato | Xi'an | Jul.11.2013 | ﹣ | <0.3 |  |
| 184 | XAJ1941 | Cucumber | Xi'an | Jul.11.2013 | ﹣ | <0.3 |  |
| 185 | HEBC1963 | Lettuce | Harbin | Jul.18.2013 | ＋ | 2 | 1963-1LM 1963-3LM |
| 186 | HEBC1964 | Coriander | Harbin | Jul.18.2013 | ﹣ | <0.3 |  |
| 187 | HEBC1965 | Cucumber | Harbin | Jul.18.2013 | ＋ | 0.62 | 1965-1LM |
| 188 | HEBJ1990 | Tomato | Harbin | Jul.18.2013 | ﹣ | <0.3 |  |
| 189 | HEBJ1991 | Cucumber | Harbin | Jul.18.2013 | ﹣ | <0.3 |  |
| 190 | JNC2013 | Tomato | Ji'nan | Jul.28.2013 | ＋ | 1.1 | 2013-1LM |
| 191 | JNC2014 | Cucumber | Ji'nan | Jul.28.2013 | ﹣ | <0.3 |  |
| 192 | JNC2015 | Tomato | Ji'nan | Jul.28.2013 | ﹣ | <0.3 |  |
| 193 | JNJ2031 | Coriander | ji'nan | Jul.28.2013 | ﹣ | <0.3 |  |
| 194 | JNJ2046 | Cucumber | Ji'nan | Jul.28.2013 | ﹣ | <0.3 |  |
| 195 | BJC2063 | Tomato | Beijing | Aug.7.2013 | ﹣ | <0.3 |  |
| 196 | BJC2064 | Coriander | Beijing | Aug.7.2013 | ﹣ | <0.3 |  |
| 197 | BJC2065 | Cucumber | Beijing | Aug.7.2013 | ﹣ | <0.3 |  |
| 198 | BJJ2090 | Tomato | Beijing | Aug.7.2013 | ﹣ | <0.3 |  |
| 199 | BJJ2091 | Cucumber | Beijing | Aug.7.2013 | ﹣ | <0.3 |  |
| 200 | TYC2113 | Tomato | Taiyuan | Aug.18.2013 | ﹣ | <0.3 |  |
| 201 | TYC2114 | Coriander | Taiyuan | Aug.18.2013 | ＋ | 0.3 | 2114-1LM 2114-3LM |
| 202 | TYC2115 | Cucumber | Taiyuan | Aug.18.2013 | ﹣ | <0.3 |  |
| 203 | TYJ2140 | Tomato | Taiyuan | Aug.18.2013 | ﹣ | <0.3 |  |
| 204 | TYJ2141 | Cucumber | Taiyuan | Aug.18.2013 | ﹣ | <0.3 |  |
| 205 | LZC2163 | Lettuce | Lanzhou | Aug.31.2013 | ﹣ | <0.3 |  |
| 206 | LZC2164 | Cucumber | Lanzhou | Aug.31.2013 | ＋ | 2.3 | 2164-2LM |
| 207 | LZC2165 | Tomato | Lanzhou | Aug.31.2013 | ﹣ | <0.3 |  |
| 208 | LZJ2181 | Coriander | Lanzhou | Aug.31.2013 | ﹣ | <0.3 |  |
| 209 | LZJ2196 | Cucumber | Lanzhou | Aug.31.2013 | ﹣ | <0.3 |  |
| 210 | FZC2213 | Tomato | Fuzhou | Nov.03.2013 | ﹣ | <0.3 |  |
| 211 | FZC2214 | Lettuce | Fuzhou | Nov.03.2013 | ﹣ | <0.3 |  |
| 212 | FZC2215 | Cucumber | Fuzhou | Nov.03.2013 | ﹣ | <0.3 |  |
| 213 | FZJ2240 | Tomato | Fuzhou | Nov.03.2013 | ﹣ | <0.3 |  |
| 214 | FZJ2241 | Cucumber | Fuzhou | Nov.03.2013 | ﹣ | <0.3 |  |
| 215 | NNC2263 | Tomato | Nanning | Nov.15.2013 | ﹣ | <0.3 |  |
| 216 | NNC2264 | Coriander | Nanning | Nov.15.2013 | ﹣ | <0.3 |  |
| 217 | NNC2265 | Cucumber | Nanning | Nov.15.2013 | ﹣ | <0.3 |  |
| 218 | NNJ2290 | Tomato | Nanning | Nov.15.2013 | ﹣ | <0.3 |  |
| 219 | NNJ2291 | Cucumber | Nanning | Nov.15.2013 | ﹣ | <0.3 |  |
| 220 | XMC2313 | Tomato | Xiamen | Nov.27.2013 | ﹣ | <0.3 |  |
| 221 | XMC2314 | Coriander | Xiamen | Nov.27.2013 | ﹣ | <0.3 |  |
| 222 | XMC2315 | Cucumber | Xiamen | Nov.27.2013 | ﹣ | <0.3 |  |
| 223 | XMJ2340 | Tomato | Xiamen | Nov.27.2013 | ﹣ | <0.3 |  |
| 224 | XMJ2341 | Cucumber | Xiamen | Nov.27.2013 | ﹣ | <0.3 |  |
| 225 | BHC2363 | Tomato | Beihai | Dec.8.2013 | ＋ | 0.61 | 2363-1LM 2363-3LM |
| 226 | BHC2364 | Cucumber | Beihai | Dec.8.2013 | ﹣ | <0.3 |  |
| 227 | BHC2365 | Cucumber | Beihai | Dec.8.2013 | ﹣ | <0.3 |  |
| 228 | BHJ2390 | Tomato | Beihai | Dec.8.2013 | ﹣ | <0.3 |  |
| 229 | BHJ2391 | Cucumber | Beihai | Dec.8.2013 | ﹣ | <0.3 |  |
| 230 | HKC2413 | Tomato | Haikou | Dec.22.2013 | ﹣ | <0.3 |  |
| 231 | HKC2414 | Tomato | Haikou | Dec.22.2013 | ﹣ | <0.3 |  |
| 232 | HKC2415 | Cucumber | Haikou | Dec.22.2013 | ﹣ | <0.3 |  |
| 233 | HKJ2440 | Tomato | Haikou | Dec.22.2013 | ﹣ | <0.3 |  |
| 234 | HKJ2441 | Cucumber | Haikou | Dec.22.2013 | ﹣ | <0.3 |  |
| 235 | SYC2463 | Tomato | Sanya | Jan.5.2014 | ﹣ | <0.3 |  |
| 236 | SYC2464 | Coriander | Sanya | Jan.5.2014 | ﹣ | <0.3 |  |
| 237 | SYC2465 | Cucumber | Sanya | Jan.5.2014 | ﹣ | <0.3 |  |
| 238 | SYJ2490 | Lettuce | Sanya | Jan.5.2014 | ﹣ | <0.3 |  |
| 239 | SYJ2491 | Cucumber | Sanya | Jan.5.2014 | ﹣ | <0.3 |  |
| 240 | NCC2513 | Tomato | Nanchang | Mar.2.2014 | ﹣ | <0.3 |  |
| 241 | NCC2514 | Cucumber | Nanchang | Mar.2.2014 | ﹣ | <0.3 |  |
| 242 | NCC2515 | Cucumber | Nanchang | Mar.2.2014 | ＋ | 0.61 | 2515-1LM 2515-2LM |
| 243 | NCJ2540 | Tomato | Nanchang | Mar.2.2014 | ﹣ | <0.3 |  |
| 244 | NCJ2541 | Cucumber | Nanchang | Mar.2.2014 | ﹣ | <0.3 |  |
| 245 | CDC2563 | Tomato | Chengdu | Mar.12.2014 | ﹣ | <0.3 |  |
| 246 | CDC2564 | Coriander | Chengdu | Mar.12.2014 | ﹣ | <0.3 |  |
| 247 | CDC2565 | Cucumber | Chengdu | Mar.12.2014 | ﹣ | <0.3 |  |
| 248 | CDJ2590 | Tomato | Chengdu | Mar.12.2014 | ﹣ | <0.3 |  |
| 249 | CDJ2591 | Cucumber | Chengdu | Mar.12.2014 | ﹣ | <0.3 |  |
| 250 | HFC2613 | Tomato | Hefei | Mar.23.2014 | ﹣ | <0.3 |  |
| 251 | HFC2614 | Cucumber | Hefei | Mar.23.2014 | ﹣ | <0.3 |  |
| 252 | HFC2615 | Cucumber | Hefei | Mar.23.2014 | ﹣ | <0.3 |  |
| 253 | HFJ2640 | Tomato | Hefei | Mar.23.2014 | ﹣ | <0.3 |  |
| 254 | HFJ2641 | Cucumber | Hefei | Mar.23.2014 | ﹣ | <0.3 |  |
| 255 | WHC2663 | Tomato | Wuhan | Apr.10.2014 | ﹣ | <0.3 |  |
| 256 | WHC2664 | Cucumber | Wuhan | Apr.10.2014 | ﹣ | <0.3 |  |
| 257 | WHC2665 | Cucumber | Wuhan | Apr.10.2014 | ﹣ | <0.3 |  |
| 258 | WHJ2690 | Tomato | Wuhan | Apr.10.2014 | ﹣ | <0.3 |  |
| 259 | WHJ2691 | Cucumber | Wuhan | Apr.10.2014 | ﹣ | <0.3 |  |
| 260 | SHC2713 | Lettuce | Shanghai | Apr.23.2014 | ﹣ | <0.3 |  |
| 261 | SHC2714 | Cucumber | Shanghai | Apr.23.2014 | ﹣ | <0.3 |  |
| 262 | SHC2715 | Cucumber | Shanghai | Apr.23.2014 | ﹣ | <0.3 |  |
| 263 | SHJ2740 | Tomato | Shanghai | Apr.23.2014 | ﹣ | <0.3 |  |
| 264 | SHJ2741 | Cucumber | Shanghai | Apr.23.2014 | ＋ | 0.3 | 2741-1LM 2741-3LM |
| 265 | KMC2763 | Lettuce | Kunming | May.6.2014 | ﹣ | <0.3 |  |
| 266 | KMC2764 | Cucumber | Kunming | May.6.2014 | ﹣ | <0.3 |  |
| 267 | KMC2765 | Cucumber | Kunming | May.6.2014 | ﹣ | <0.3 |  |
| 268 | KMJ2790 | Tomato | Kunming | May.6.2014 | ﹣ | <0.3 |  |
| 269 | KMJ2791 | Cucumber | Kunming | May.6.2014 | ﹣ | <0.3 |  |
| 270 | CSC2813 | Tomato | Changsha | Jun.30.2015 | ﹣ | <0.3 |  |
| 271 | CSC2814 | Coriander | Changsha | Jun.30.2015 | ﹣ | <0.3 |  |
| 272 | CSC2815 | Cucumber | Changsha | Jun.30.2015 | ﹣ | 0.36 |  |
| 273 | CSJ2840 | Tomato | Changsha | Jun.30.2015 | ﹣ | <0.3 |  |
| 274 | CSJ2841 | Cucumber | Changsha | Jun.30.2015 | ﹣ | <0.3 |  |
| 275 | HZC2863 | Tomato | Hangzhou | Jul.17.2015 | ﹣ | <0.3 |  |
| 276 | HZC2864 | Coriander | Hangzhou | Jul.17.2015 | ﹣ | <0.3 |  |
| 277 | HZC2865 | Cucumber | Hangzhou | Jul.17.2015 | ﹣ | <0.3 |  |
| 278 | HZJ2890 | Tomato | Hangzhou | Jul.17.2015 | ﹣ | <0.3 |  |
| 279 | HZJ2891 | Cucumber | Hangzhou | Jul.17.2015 | ﹣ | <0.3 |  |
| 280 | GYC2913 | Tomato | Guiyang | Jul.26.2015 | ﹣ | <0.3 |  |
| 281 | GYC2914 | Coriander | Guiyang | Jul.26.2015 | ﹣ | <0.3 |  |
| 282 | GYC2915 | Cucumber | Guiyang | Jul.26.2015 | ﹣ | <0.3 |  |
| 283 | GYJ2940 | Tomato | Guiyang | Jul.26.2015 | ﹣ | <0.3 |  |
| 284 | GYJ2941 | Cucumber | Guiyang | Jul.26.2015 | ﹣ | <0.3 |  |
| 285 | CCC2963 | Tomato | Changchun | Aug.4.2015 | ﹣ | <0.3 |  |
| 286 | CCC2964 | Coriander | Changchun | Aug.4.2015 | ﹣ | <0.3 |  |
| 287 | CCC2965 | Cucumber | Changchun | Aug.4.2015 | ﹣ | <0.3 |  |
| 288 | CCJ2990 | Tomato | Changchun | Aug.4.2015 | ﹣ | <0.3 |  |
| 289 | CCJ2991 | Cucumber | Changchun | Aug.4.2015 | ﹣ | <0.3 |  |
| 290 | XNC3013 | Tomato | Xi'ning | Aug.19.2015 | ﹣ | <0.3 |  |
| 291 | XNC3014 | Coriander | xi'ning | Aug.19.2015 | ﹣ | <0.3 |  |
| 292 | XNC3015 | Cucumber | Xi'ning | Aug.19.2015 | ＋ | 0.3 | 3015-1LM |
| 293 | XNJ3040 | Tomato | Xi'ning | Aug.19.2015 | ﹣ | <0.3 |  |
| 294 | XNJ3041 | Cucumber | Xi'ning | Aug.19.2015 | ﹣ | <0.3 |  |
| 295 | YCC3063 | Tomato | Yinchuan | Aug.26.2015 | ﹣ | <0.3 |  |
| 296 | YCC3064 | Coriander | Yinchuan | Aug.26.2015 | ﹣ | <0.3 |  |
| 297 | YCC3065 | Cucumber | Yinchuan | Aug.26.2015 | ﹣ | <0.3 |  |
| 298 | YCJ3090 | Tomato | Yinchuan | Aug.26.2015 | ﹣ | <0.3 |  |
| 299 | YCJ3091 | Cucumber | Yinchuan | Aug.26.2015 | ﹣ | <0.3 |  |
| 300 | HHHTC3113 | Tomato | Hohhot | Sep.8.2015 | ﹣ | <0.3 |  |
| 301 | HHHTC3114 | Coriander | Hohhot | Sep.8.2015 | ﹣ | <0.3 |  |
| 302 | HHHTC3115 | Cucumber | Hohhot | Sep.8.2015 | ﹣ | <0.3 |  |
| 303 | HHHTJ3140 | Tomato | Hohhot | Sep.8.2015 | ﹣ | <0.3 |  |
| 304 | HHHTJ3141 | Cucumber | Hohhot | Sep.8.2015 | ﹣ | <0.3 |  |
| 305 | SYC3163 | Tomato | Shenyang | Sep.14.2015 | ﹣ | <0.3 |  |
| 306 | SYC3164 | Coriander | Shenyang | Sep.14.2015 | ﹣ | <0.3 |  |
| 307 | SYC3165 | Cucumber | Shenyang | Sep.14.2015 | ﹣ | <0.3 |  |
| 308 | SYJ3190 | Tomato | Shenyang | Sep.14.2015 | ﹣ | <0.3 |  |
| 309 | SYJ3191 | Cucumber | Shenyang | Sep.14.2015 | ﹣ | <0.3 |  |
| 310 | NJC3213 | Lettuce | Nanjing | Sep.21.2015 | ﹣ | <0.3 |  |
| 311 | NJC3214 | Tomato | Nanjing | Sep.21.2015 | ﹣ | <0.3 |  |
| 312 | NJC3215 | Cucumber | Nanjing | Sep.21.2015 | ﹣ | <0.3 |  |
| 313 | NJJ3240 | Lettuce | Nanjing | Sep.21.2015 | ﹣ | <0.3 |  |
| 314 | NJJ3241 | Cucumber | Nanjing | Sep.21.2015 | ﹣ | <0.3 |  |
| 315 | SJZC3263 | Lettuce | Shijiazhuang | Oct.09.2015 | ﹣ | <0.3 |  |
| 316 | SJZC3264 | Tomato | Shijiazhuang | Oct.09.2015 | ﹣ | <0.3 |  |
| 317 | SJZC3265 | Cucumber | Shijiazhuang | Oct.09.2015 | ﹣ | <0.3 |  |
| 318 | SJZJ3290 | Lettuce | Shijiazhuang | Oct.09.2015 | ﹣ | <0.3 |  |
| 319 | SJZJ3291 | Cucumber | Shijiazhuang | Oct.09.2015 | ﹣ | <0.3 |  |
| 320 | ZZC3313 | Tomato | Zhengzhou | Oct.21.2015 | ﹣ | <0.3 |  |
| 321 | ZZC3314 | Coriander | Zhengzhou | Oct.21.2015 | ﹣ | <0.3 |  |
| 322 | ZZC3315 | Cucumber | Zhengzhou | Oct.21.2015 | ﹣ | <0.3 |  |
| 323 | ZZJ3340 | Tomato | Zhengzhou | Oct.21.2015 | ＋ | 24 | 3340-2LM |
| 324 | ZZJ3341 | Lettuce | Zhengzhou | Oct.21.2015 | ﹣ | <0.3 |  |
| 325 | LSC3363 | Tomato | Lhasa | Nov.14.2015 | ﹣ | <0.3 |  |
| 326 | LSC3364 | Coriander | Lhasa | Nov.14.2015 | ﹣ | <0.3 |  |
| 327 | LSC3365 | Cucumber | Lhasa | Nov.14.2015 | ﹣ | <0.3 |  |
| 328 | LSJ3390 | Tomato | Lhasa | Nov.14.2015 | ﹣ | <0.3 |  |
| 329 | LSJ3391 | Lettuce | Lhasa | Nov.14.2015 | ﹣ | <0.3 |  |
| 331 | WLMQC3413 | Tomato | Urumchi | Nov.23.2015 | ﹣ | <0.3 |  |
| 332 | WLMQC3414 | Coriander | Urumchi | Nov.23.2015 | ﹣ | <0.3 |  |
| 333 | WLMQC3415 | Cucumber | Urumchi | Nov.23.2015 | ﹣ | <0.3 |  |
| 334 | WLMQJ3440 | Tomato | Urumchi | Nov.23.2015 | ﹣ | <0.3 |  |
| 335 | WLMQJ3441 | Coriander | Urumchi | Nov.23.2015 | ﹣ | <0.3 |  |
| 336 | AMC3463 | Tomato | Macao | Dec.8.2015 | ﹣ | <0.3 |  |
| 337 | AMC3464 | Cucumber | Macao | Dec.8.2015 | ﹣ | <0.3 |  |
| 338 | AMC3465 | Cucumber | Macao | Dec.8.2015 | ﹣ | <0.3 |  |
| 339 | AMJ3490 | Tomato | Macao | Dec.8.2015 | ﹣ | <0.3 |  |
| 330 | AMJ3491 | Lettuce | Macao | Dec.8.2015 | ＋ | 2.1 | 3491-3LM |
| 340 | HKC3513 | Tomato | Hongkong | Dec.22.2015 | ﹣ | <0.3 |  |
| 341 | HKC3514 | Coriander | Hongkong | Dec.22.2015 | ﹣ | <0.3 |  |
| 342 | HKC3515 | Cucumber | Hongkong | Dec.22.2015 | ﹣ | <0.3 |  |
| 343 | HKJ3540 | Tomato | Hongkong | Dec.22.2015 | ﹣ | <0.3 |  |
| 344 | HKJ3541 | Cucumber | Hongkong | Dec.22.2015 | ﹣ | <0.3 |  |
| 345 | CSC3563 | Tomato | Changsha | Jan.11.2016 | ﹣ | <0.3 |  |
| 346 | CSC3564 | Cucumber | Changsha | Jan.11.2016 | ﹣ | <0.3 |  |
| 347 | CSC3565 | Lettuce | Changsha | Jan.11.2016 | ﹣ | <0.3 |  |
| 348 | CSJ3590 | Tomato | Changsha | Jan.11.2016 | ﹣ | <0.3 |  |
| 349 | CSJ3591 | Lettuce | Changsha | Jan.11.2016 | ﹣ | <0.3 |  |
| 350 | GYC3613 | Tomato | Guiyang | Jan.20.2016 | ﹣ | <0.3 |  |
| 351 | GYC3614 | Cucumber | Guiyang | Jan.20.2016 | ﹣ | <0.3 |  |
| 352 | GYC3615 | Lettuce | Guiyang | Jan.20.2016 | ﹣ | <0.3 |  |
| 353 | GYJ3640 | Tomato | Guiyang | Jan.20.2016 | ﹣ | <0.3 |  |
| 354 | GYJ3641 | Lettuce | Guiyang | Jan.20.2016 | ﹣ | <0.3 |  |
| 355 | HZC3663 | Tomato | Hangzhou | Feb.24.2016 | ﹣ | <0.3 |  |
| 356 | HZC3664 | Cucumber | Hangzhou | Feb.24.2016 | ﹣ | <0.3 |  |
| 357 | HZC3665 | Lettuce | Hangzhou | Feb.24.2016 | ＋ | <0.3 | 3665-1LM |
| 358 | HZJ3690 | Tomato | Hangzhou | Feb.24.2016 | ﹣ | <0.3 |  |
| 359 | HZJ3691 | Lettuce | Hangzhou | Feb.24.2016 | ﹣ | <0.3 |  |
| 360 | NJC3713 | Tomato | Nanjing | Mar.2.2016 | ﹣ | <0.3 |  |
| 361 | NJC3714 | Cucumber | Nanjing | Mar.2.2016 | ﹣ | <0.3 |  |
| 362 | NJC3715 | Lettuce | Nanjing | Mar.2.2016 | ﹣ | <0.3 |  |
| 363 | NJJ3740 | Tomato | Nanjing | Mar.2.2016 | ﹣ | <0.3 |  |
| 364 | NJJ3741 | Cucumber | Nanjing | Mar.2.2016 | ﹣ | <0.3 |  |
| 365 | SJZC3763 | Tomato | Shijiazhuang | Mar.9.2016 | ﹣ | <0.3 |  |
| 366 | SJZC3764 | Cucumber | Shijiazhuang | Mar.9.2016 | ﹣ | <0.3 |  |
| 367 | SJZC3765 | Lettuce | Shijiazhuang | Mar.9.2016 | ﹣ | <0.3 |  |
| 368 | SJZJ3790 | Coriander | Shijiazhuang | Mar.9.2016 | ﹣ | <0.3 |  |
| 369 | SJZJ3791 | Lettuce | Shijiazhuang | Mar.9.2016 | ﹣ | <0.3 |  |
| 370 | SYC3813 | Tomato | Shenyang | Mar.18.2016 | ﹣ | <0.3 |  |
| 371 | SYC3814 | Cucumber | Shenyang | Mar.18.2016 | ﹣ | <0.3 |  |
| 372 | SYC3815 | Lettuce | Shenyang | Mar.18.2016 | ﹣ | <0.3 |  |
| 373 | SYJ3840 | Coriander | Shenyang | Mar.18.2016 | ﹣ | <0.3 |  |
| 374 | SYJ3841 | Lettuce | Shenyang | Mar.18.2016 | ﹣ | <0.3 |  |
| 375 | YCC3863 | Tomato | Yinchuan | Mar.28.2016 | ﹣ | <0.3 |  |
| 376 | YCC3864 | Cucumber | Yinchuan | Mar.28.2016 | ﹣ | <0.3 |  |
| 377 | YCC3865 | Lettuce | Yinchuan | Mar.28.2016 | ﹣ | <0.3 |  |
| 378 | YCJ3890 | Coriander | Yinchuan | Mar.28.2016 | ﹣ | <0.3 |  |
| 379 | YCJ3891 | Lettuce | Yinchuan | Mar.28.2016 | ﹣ | <0.3 |  |
| 380 | ZZC3913 | Tomato | Zhengzhou | Apr.6.2016 | ﹣ | <0.3 |  |
| 381 | ZZC3914 | Cucumber | Zhengzhou | Apr.6.2016 | ﹣ | <0.3 |  |
| 382 | ZZC3915 | Lettuce | Zhengzhou | Apr.6.2016 | ﹣ | <0.3 |  |
| 383 | ZZJ3940 | Coriander | Zhengzhou | Apr.6.2016 | ＋ | 0.3 | 3940-1LM |
| 384 | ZZJ3941 | Lettuce | Zhengzhou | Apr.6.2016 | ﹣ | <0.3 |  |
| 385 | XNC3963 | Tomato | Xi'ning | Apr.13.2016 | ﹣ | <0.3 |  |
| 386 | XNC3964 | Cucumber | Xi'ning | Apr.13.2016 | ﹣ | <0.3 |  |
| 387 | XNC3965 | Lettuce | Xi'ning | Apr.13.2016 | ﹣ | <0.3 |  |
| 388 | XNJ3990 | Coriander | Xi'ning | Apr.13.2016 | ﹣ | <0.3 |  |
| 389 | XNJ3991 | Lettuce | Xi'ning | Apr.13.2016 | ﹣ | <0.3 |  |
| 390 | HHHTC4013 | Tomato | Hohhot | Apr.21.2016 | ﹣ | <0.3 |  |
| 391 | HHHTC4014 | Cucumber | Hohhot | Apr.21.2016 | ﹣ | <0.3 |  |
| 392 | HHHTC4015 | Lettuce | Hohhot | Apr.21.2016 | ﹣ | <0.3 |  |
| 393 | HHHTJ4040 | Coriander | Hohhot | Apr.21.2016 | ﹣ | <0.3 |  |
| 396 | HHHTJ4041 | Lettuce | Hohhot | Apr.21.2016 | ﹣ | <0.3 |  |
| 394 | CCC4063 | Tomato | Changchun | May.4.2016 | ﹣ | <0.3 |  |
| 397 | CCC4064 | Cucumber | Changchun | May.4.2016 | ﹣ | <0.3 |  |
| 398 | CCC4065 | Coriander | Changchun | May.4.2016 | ﹣ | <0.3 |  |
| 399 | CCJ4090 | Coriander | Changchun | May.4.2016 | ﹣ | <0.3 |  |
| 400 | CCJ4091 | Cucumber | Changchun | May.4.2016 | ﹣ | <0.3 |  |
| 401 | AMC4113 | Coriander | Macao | Jul.11.2016 | ﹣ | <0.3 |  |
| 402 | AMC4114 | Cucumber | Macao | Jul.11.2016 | ﹣ | <0.3 |  |
| 403 | AMC4115 | Cucumber | Macao | Jul.11.2016 | ﹣ | <0.3 |  |
| 395 | AMJ4140 | Tomato | Macao | Jul.11.2016 | ﹣ | <0.3 |  |
| 404 | AMJ4141 | Cucumber | Macao | Jul.11.2016 | ﹣ | <0.3 |  |
| 405 | XGC4163 | Tomato | Hongkong | May.31.2016 | ﹣ | <0.3 |  |
| 406 | XGC4164 | Coriander | Hongkong | May.31.2016 | ﹣ | <0.3 |  |
| 407 | XGC4165 | Cucumber | Hongkong | May.31.2016 | ﹣ | <0.3 |  |
| 408 | XGJ4190 | Tomato | Hongkong | May.31.2016 | ﹣ | <0.3 |  |
| 409 | XGJ4191 | Cucumber | Hongkong | May.31.2016 | ﹣ | <0.3 |  |
| 410 | WLMQC4213 | Tomato | Urumchi | Jun.16.2016 | ﹣ | <0.3 |  |
| 411 | WLMQC4214 | Lettuce | Urumchi | Jun.16.2016 | ﹣ | <0.3 |  |
| 412 | WLMQC4215 | Cucumber | Urumchi | Jun.16.2016 | ﹣ | <0.3 |  |
| 413 | WLMQJ4240 | Tomato | Urumchi | Jun.16.2016 | ﹣ | <0.3 |  |
| 414 | WLMQJ4241 | Cucumber | Urumchi | Jun.16.2016 | ﹣ | <0.3 |  |
| 415 | LSC4263 | Tomato | Lhasa | Jun.23.2016 | ﹣ | <0.3 |  |
| 416 | LSC4264 | Coriander | Lhasa | Jun.23.2016 | ﹣ | <0.3 |  |
| 417 | LSC4265 | Cucumber | Lhasa | Jun.23.2016 | ﹣ | <0.3 |  |
| 418 | LSJ4290 | Tomato | Lhasa | Jun.23.2016 | ﹣ | <0.3 |  |
| 419 | LSJ4291 | Lettuce | Lhasa | Jun.23.2016 | ﹣ | <0.3 |  |

| **Table S2 *Listeria monocytogenes* strains isolated from fresh vegetables** | | | | | | | |
| --- | --- | --- | --- | --- | --- | --- | --- |
| Numbers | Strains | Numbers | Strains | Numbers | Strains | Numbers | Strains |
| 1 | 133-1  LM | 9 | 1741-1  LM | 17 | 2114-1  LM | 25 | 2741-3  LM |
| 2 | 413-2  LM | 10 | 1765-1  LM | 18 | 2114-3  LM | 26 | 3015-1  LM |
| 3 | 413-4  LM | 11 | 1791-1  LM | 19 | 2164-2  LM | 27 | 3340-2  LM |
| 4 | 1231-1  LM | 12 | 1791-2  LM | 20 | 2363-1  LM | 28 | 3491-3  LM |
| 5 | 1263-1  LM | 13 | 1963-1  LM | 21 | 2363-3  LM | 29 | 3665-1  LM |
| 6 | 1331-1  LM | 14 | 1963-3  LM | 22 | 2515-1  LM | 30 | 3940-1  LM |
| 7 | 1331-2  LM | 15 | 1965-1  LM | 23 | 2515-2  LM |  |  |
| 8 | 1614-1  LM | 16 | 2013-1  LM | 24 | 2741-1  LM |  |  |

**Figure S1. Serogroup analysis of *Listeria monocytogenes* strains isolated from fresh vegetable samples by multiplex PCR.**

The strain no. 1-30 correspond to Table S2.

**Figure S2. The presence of virulence-related genes in *Listeria monocytogenes* isolated from fresh vegetable samples**.

A, *prfA*; B, *mpl*; C, *plcA*; D, *inlB*; E, *plcA*; F, *hly*; G, *iap*; H, *actA*; I, *llsX*; J, *ptsA*.*:The strain no. 1-30 correspond to Table S2.
