# Supplementary figures and images for: Genetic characteristics and virulence of Listeria monocytogenes isolated from fresh vegetables in China
Source: BMC Microbiol. 2019 Jun 3;19:119. doi: 10.1186/s12866-019-1488-5 (PMC6547522; doi:10.1186/s12866-019-1488-5)

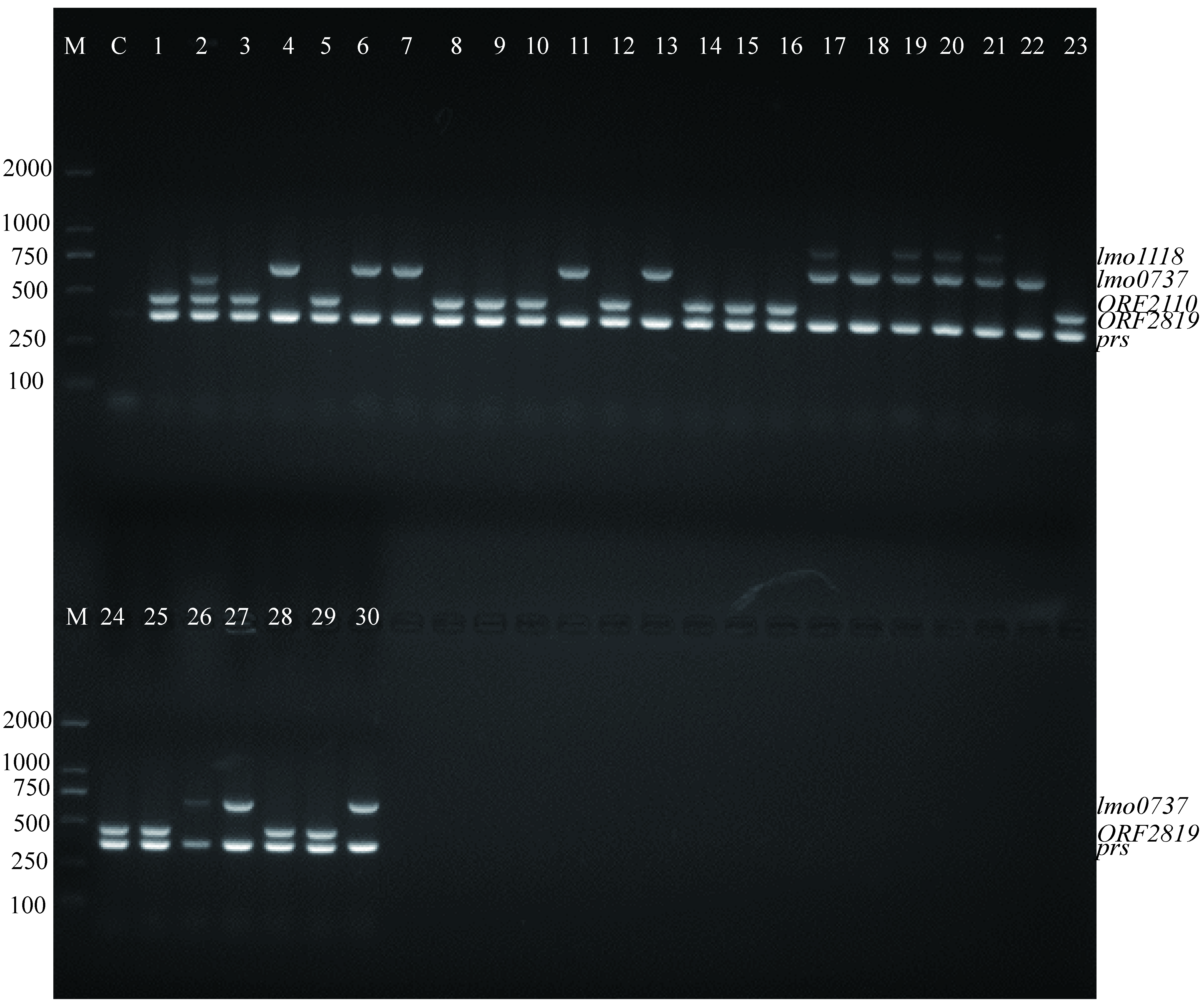

Supplement: Supplementary file 1 — Table S1. The detail detection results of Listeria monocytogenes in 419 vegetable samples. Table S2. Listeria monocytogenes strains isolated from fresh vegetables Figure S1. Serogroup analysis of Listeria monocytogenes strains isolated from fresh vegetable samples by multiplex PCR. The strain no. 1–30 correspond to Table S2. Figure S2. The presence of virulence-related genes in Listeria monocytogenes isolated from fresh vegetable samples. A, prfA; B, mpl; C, plcA; D, inlB; E, plcA; F, hly; G, iap; H, actA; I, llsX; J, ptsA.*:The strain no. 1–30 correspond to Additional file 1: Table S2. (ZIP 8979 kb) [file 12866_2019_1488_MOESM1_ESM.zip › Figure S1.TIF.tif]

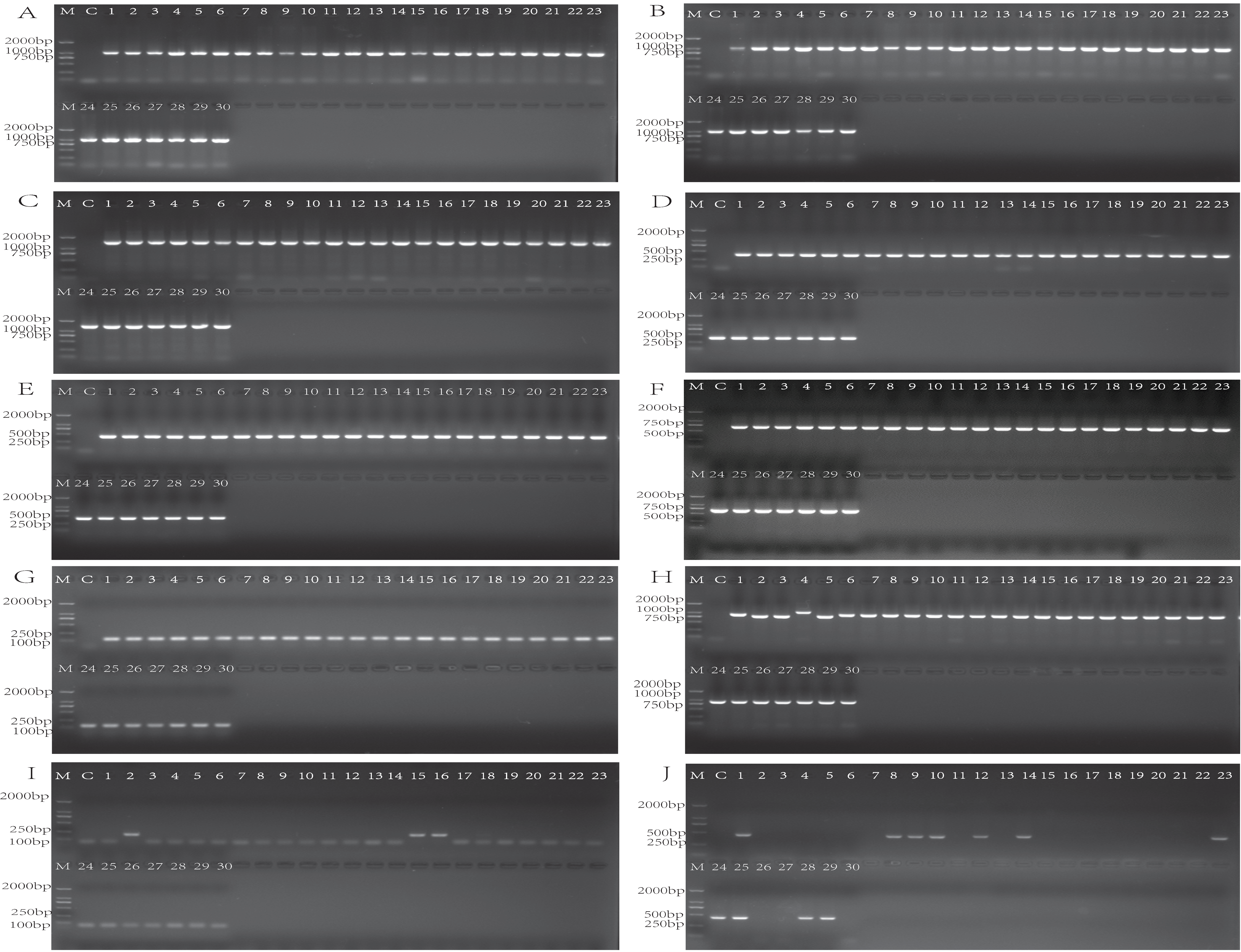

Supplement: Supplementary file 1 — Table S1. The detail detection results of Listeria monocytogenes in 419 vegetable samples. Table S2. Listeria monocytogenes strains isolated from fresh vegetables Figure S1. Serogroup analysis of Listeria monocytogenes strains isolated from fresh vegetable samples by multiplex PCR. The strain no. 1–30 correspond to Table S2. Figure S2. The presence of virulence-related genes in Listeria monocytogenes isolated from fresh vegetable samples. A, prfA; B, mpl; C, plcA; D, inlB; E, plcA; F, hly; G, iap; H, actA; I, llsX; J, ptsA.*:The strain no. 1–30 correspond to Additional file 1: Table S2. (ZIP 8979 kb) [file 12866_2019_1488_MOESM1_ESM.zip › Figure S2.TIF.tif]
